# Aging with Cerebral Small Vessel Disease and Dizziness: The Importance of Undiagnosed Peripheral Vestibular Disorders

**DOI:** 10.3389/fneur.2017.00241

**Published:** 2017-06-02

**Authors:** Niccolò Cerchiai, Michelangelo Mancuso, Elena Navari, Nicola Giannini, Augusto Pietro Casani

**Affiliations:** ^1^Department of Medical and Surgical Pathology, Otorhinolaryngology Section, Pisa University, Pisa, Italy; ^2^Department of Experimental and Clinical Medicine, Neurological Institute, Pisa University, Pisa, Italy

**Keywords:** vertigo, dizziness, leukoaraiosis, vestibular loss, benign paroxysmal vertigo, white matter hyperintensities, aging, falls

## Abstract

Recent studies showed a link between cerebral small vessel white matter disease (SVD) and dizziness: patients whose dizziness cannot be explained by vestibular disease show severe SVD and gait abnormalities; however, little is still known about how SVD can cause this symptom. The primary aim of this study is to examine the possible underlying causes of dizziness in neurovascular patients; this is in order to assess whether treatable causes could be routinely disregarded. A secondary aim is to possibly define a central oculomotor pattern induced *per se* by SVD. This could help the diagnosis of SVD-related dizziness. In this single-blind prospective study, 60 patients referred to a neurovascular clinic because of dizziness and SVD on imaging were divided into an L-SVD and a H-SVD group (low and high SVD burden, respectively), and then blindly examined with vestibulometric tests. In H-SVD group, the percentage of unexplained dizziness reached 82.8%. There was a higher prevalence of peripheral vestibular abnormalities in the L-SVD patient group (51.6%) than in the H-SVD (17.2%; *p* = 0.012). We found no differences in central oculomotor findings between the two groups. Although oculomotricity does not show any consistent pattern, a severe SVD can directly represent a cause of dizziness. However, a patient with mild SVD is more likely to suffer by a peripheral vestibular disorder. Therefore, given the high incidence of vestibular disease in neurovascular or geriatric clinics, clinicians should be cautious when ascribing dizziness solely to the presence of SVD as easily treatable peripheral vestibular causes may be missed.

## Introduction

Cerebral small vessel white matter disease (SVD) has recently generated great interest in neurovascular practice because of its possible role in the development of geriatric syndromes (1, 2). Although it has been established how SVD contributes to the development of cognitive decline and dementia (3, 4) and falls (5, 6), little is known about SVD as a possible cause of dizziness. White matter hyperintensities on T2-weighted and fluid-attenuated inversion recovery sequences on magnetic resonance imaging (MRI) are the radiological expression of SVD and represent a well-known marker of a higher risk of cerebral, cerebellar, and brainstem stroke (7). A study by Okroglic and colleagues showed that dizziness, defined as an illusion of movement, could affect up to 17% of patients with SVD (8). In a previous study, together with the colleagues belonging to the Neurotology Unit of the Charing Cross Hospital in London, we looked at two groups of patients referred to neurotology services; one group with dizziness ascribable to definite causes (both peripheral and central vestibular disorders) and one with dizziness of an uncertain origin (unexplained dizziness). We found that SVD burden represents a significant predictor of unexplained dizziness and gait abnormalities; we postulated that white matter lesions may induce dizziness either because patients perceive a degree of objective unsteadiness or by a cortical–subcortical disconnection syndrome, secondary to disruption of white matter tracts involved in gait and balance control (9). The present study addresses the following research questions: (1) Are there any underlying causes of dizziness in elderly patients with already known SVD? This question is of great relevance to clinicians in both neurovascular and geriatric clinics as, in patients with established SVD, some potentially treatable peripheral causes of dizziness/unsteadiness may be disregarded. (2) Can an accurate neurotological assessment reveal any consistent central oculomotor pattern induced *per se* by SVD?

## Materials and Methods

In order to assess the two research questions, the study prospectively analyzed the clinical data of 60 patients referred to the Neurovascular service of a tertiary referral center (Pisa University Hospital, Italy) because of chronic dizziness and/or a degree of unsteadiness. The brain scan (1.5 or 3 T MRI) showed white matter lesions compatible with a degree of SVD. A vascular neurologist (NG) reviewed the scans and attributed a specific degree of SVD burden according to the Fazekas’ scale (7), which was in agreement with the neuroradiologist’s report. Although the reported dizziness could have been ascribed to SVD only, other possible causes had not been excluded. For this reason, the patients were then referred on to otolaryngology consultants belonging to the Neurotology service (NC, EN); a senior consultant (AC) reviewed both clinical and laboratory findings. The neurotological examination was done blindly to the MRI findings. All the patients were previously screened with the Mini Mental State Examination, in order to exclude subjects with cognitive impairment (patients scoring less than 25 were excluded), before referral to the neurotology service. Similarly, the vascular neurologists applied the following exclusion criteria: (1) major strokes or cerebral bleedings; (2) other causes of leukoencephalopathy (e.g., immune, demyelinating, metabolic, toxic, infectious, genetic); (3) severe unrelated neurological pathologies (e.g., severe neuropathy, spasticity, extrapyramidal syndromes); (4) psychiatric disorders or severe cognitive decline; (5) age greater than 90 years. Since the selection criteria excluded most of the neurological causes of explainable dizziness, patients without signs of peripheral vestibular disorders would have been likely to suffer from an unexplained dizziness.

This study was carried out in accordance with the hospital protocols. All subjects gave written informed consent in accordance with the Declaration of Helsinki.

The neurotological assessment comprised the following: detailed clinical history, pure tone audiometry, oculography through a biocular mask with integrated infrared cameras (4-View system by Synapsys, Marseille, France), head shaking test, caloric test, and video head impulse test (vHIT). Oculography was performed in order to reveal abnormalities beyond what is expected for the patient’s age: peripheral vestibular features or central oculomotor abnormalities. Caloric test was performed according to a modified Fitzgerald–Hallpike technique: the external auditory canal was irrigated with 125 cc of warm (44°C) and cold (30°C) water for 30 s (7 min elapsing between irrigations); ocular responses were recorded through an infrared eye-tracking system (GN Otometrics, Taastrup, Denmark). Canal Paresis was considered as significant if greater than 25% (Jongkees formula). The vHIT was performed with a dedicated device (“ICS Impulse” system; GN Otometrics, Taastrup, Denmark—http://www.icsimpulse.com): the patient was asked to stare at an earth-fixed target (3 cm diameter spot located 1.5 m in front); then, 20 horizontal impulses (10–20° amplitude) were randomly administered to each side. An average of the vestibulo-oculomotor reflex (VOR) gain was calculated by the device software; when equal or lower than 0.69 was considered pathological; values between 0.70 and 0.79 were considered as borderline. Both caloric test and vHIT parameters have been validated according to our own normative data on healthy subjects. We considered as signs of peripheral vestibular involvement the following: typical benign paroxysmal positional vertigo (BPPV) nystagmus, canal paresis on caloric test, and decreased gain of the VOR on vHIT. In defining central oculomotor patterns possibly induced by SVD, we considered the following vestibular and non-vestibular central oculomotor abnormalities: gaze evoked nystagmus, cross-coupled head shaking nystagmus, downbeating (or atypical) positional nystagmus, spontaneous nystagmus of a central type, broken smooth pursuit, or saccades abnormalities. The severity of dizziness-related symptoms was investigated with the Dizziness Handicap Inventory (DHI) questionnaire (10).

### Statistical Analysis

The Kolmogorov–Smirnov test and Levene’s test, were used, respectively, to assess normal distribution of data and homogeneity of variance. Differences between parametric and non-parametric data were assessed with a *t*-test for independent samples and Mann–Whitney test, respectively. We applied Chi-square test with Yates’ correction to determine differences between prevalence (frequency). Significance was set at *p* < 0.05. In order to address the research questions posed, we analyzed separately patients with SVD, by dividing them into low-grade SVD (Fazekas 0 or 1; L-SVD group) and moderate–high SVD (Fazekas 2 or 3; H-SVD group).

### Ethical Standards Statement

The study was approved by the local ethics committee and conducted in accordance with the principles outlined in the Declaration of Helsinki. All patients gave their informed consent prior to the inclusion in the study.

## Results

There were 31 patients in the L-SVD group (age range 50–83 years, mean 69.7, SD 8.0) and 29 in H-SVD (age range 49–89 years, mean 73.4, SD 8.9); these two groups showed no differences in terms of age (*t*-test; *p* = 0.10). As expected, there was a small tendency to have higher grades of SVD in older people (Fazekas 0–1, 2, and 3: mean age 69.7, 72.2, and 78.0 years, respectively).

**Figure 1:**
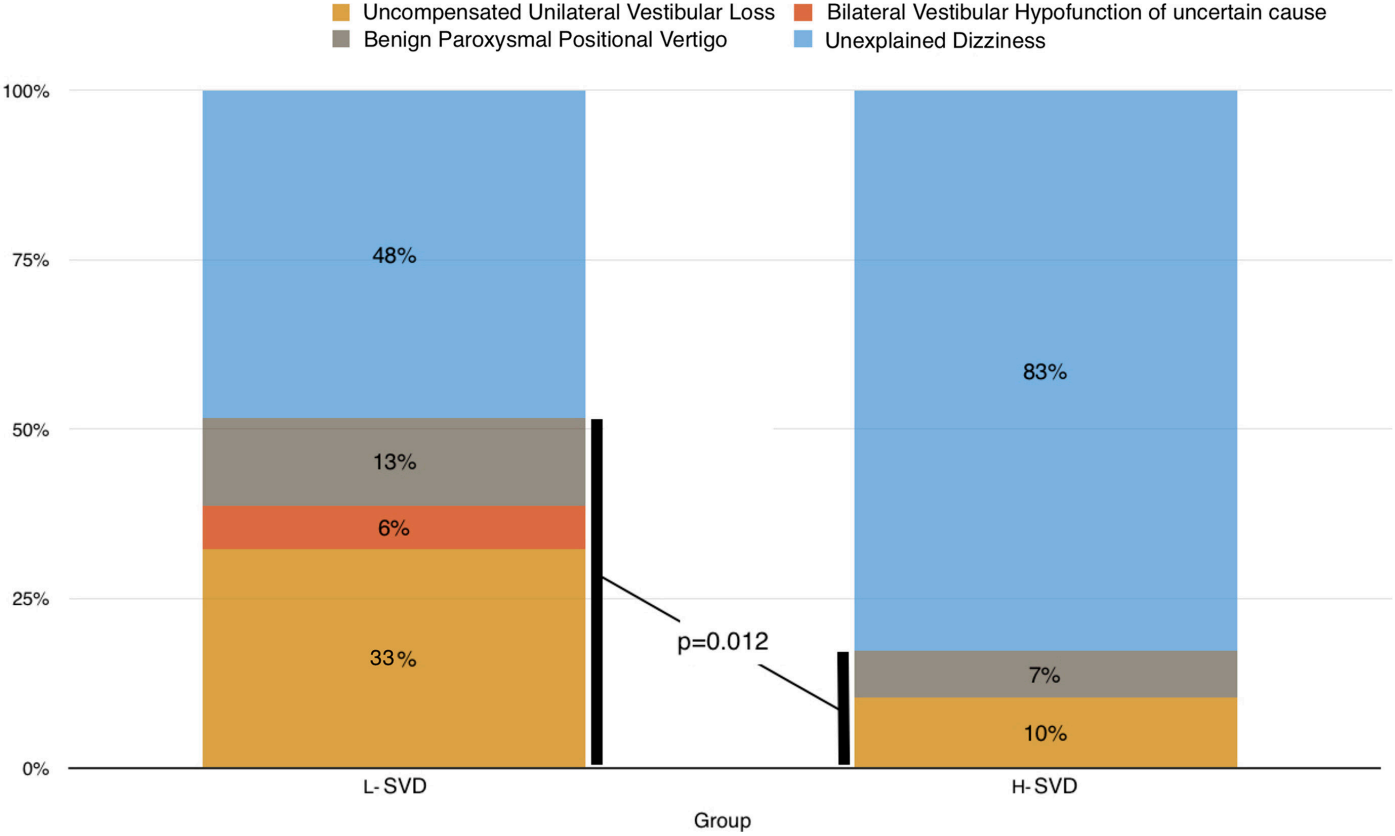
Peripheral vestibular causes of dizziness in L-SVD and H-SVD groups.

In the majority of the patients, the neurotological assessment could not allow the clinician to detect pathologies different from SVD able to explain the dizziness. In particular, the percentage of unexplained dizziness reached 82.8% in H-SVD group and 48.4% in L-SVD group (Figure 1). At the same time, the neurotological investigations revealed the presence of peripheral vestibular abnormalities, involving mostly the L-SVD group. There was a higher prevalence of pathological vestibular signs of a peripheral type in patients with lower SVD burden (51.6% in L-SVD) with respect to those with higher burden (17.2% in H-SVD; Chi-square test; *p* = 0.012). A significant canal paresis was found in 12 patients (38.7%) of L-SVD (in 2 cases a very low slow phase velocity response—1.3 and 1.8°/s, respectively—indicated a bilateral vestibular hypofunction) and in 3 patients (10.3%) of H-SVD (Figure 1). Since unilateral canal pareses were always associated with pathological vHIT, in these cases the dizziness was likely due to insufficient central compensation after a previous vestibular loss. The two patients belonging to L-SVD group and having a bilateral vestibular hypofunction did not report any exposition to vestibulo-toxic agents; since they also showed mild signs of cerebellar involvement, they were then followed up in the Neurology clinic in order to exclude the development of cerebellar ataxia or neuropathies (CANVAS syndrome). All the patients with vestibular hypofunction were then referred to a vestibular rehabilitation program. One of the most important findings of this study is represented by the percentage of unrecognized BPPVs. An undiagnosed BPPV was found in four patients (12.9%) of L-SVD and in two (6.9%) of H-SVD; these cases were then easily treated through canalith repositioning procedures. Detailed peripheral vestibulo-ocular findings are shown in Table 1.

**Table 1:** Demographic characteristics and peripheral vestibular findings in the group with low and medium–high small vessel disease burden (L-SVD and H-SVD, respectively).

		L-SVD group	H-SVD Group	*p*-Value

		*N* (%)	*N* (%)	
Demographics	Total	31 (100%)	29 (100%)	0.10
	Sex	M 20 (64.5), F 11 (35.5)	M 13 (44.8), F 16 (55.2)	
Peripheral vestibular findings^a^	Typical paroxysmal positional nystagmus	4 (12.9)	2 (6.9)	0.012
	Spontaneous nystagmus (peripheral type)	4 (12.9)	2 (6.9)	
	Canal paresis	13 (41.9)	3 (10.3)	
	Abnormal head impulse test	4 (12.9)	2 (6.9)	
	Abnormal video head impulse test	13 (41.9)	3 (10.3)	

*^a^More findings than cases are listed due to patients with multiple peripheral features*.

**Figure 2:**
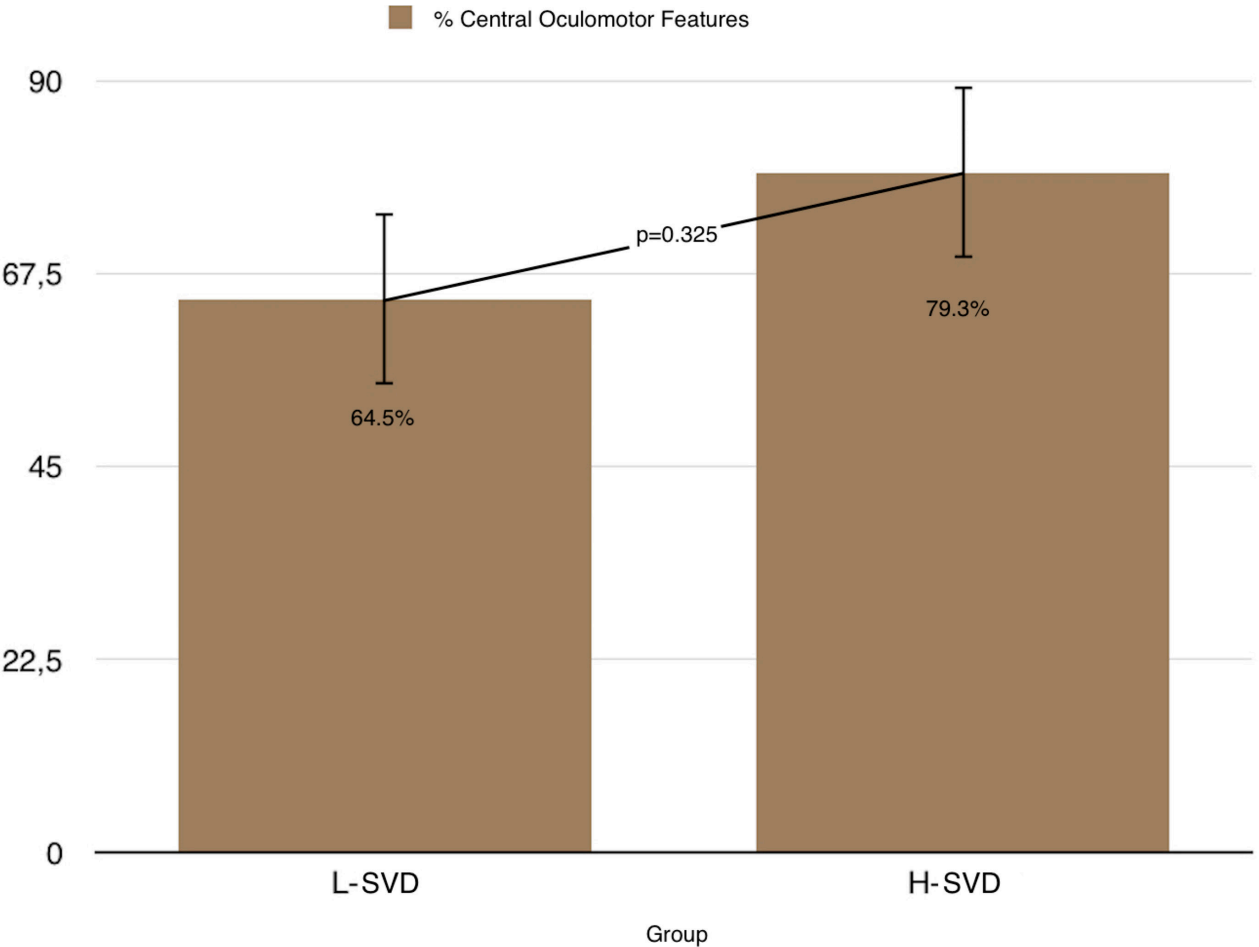
Prevalence of central oculomotor features in L-SVD and H-SVD groups.

Although in 71.6% of patients we detected minor to moderate central oculomotor abnormalities (Table 2), we were not able to identify consistent patterns. No difference between L-SVD and H-SVD was found in terms of prevalence of central oculomotor features like gaze-evoked nystagmus, cross-coupled head shaking test, downbeating (or atypical) positional nystagmus, spontaneous nystagmus of a central type, broken smooth pursuit, or minor saccades abnormalities (64.5% in L-SVD and 79.3% in H-SVD—Chi-square test; *p* = 0.325) (Figure 2). Table 3 shows in detail the number of patients with central oculomotor abnormalities among different degrees of Fazekas’ scale (Table 3).

**Table 2:** Central oculomotor features in the group with low and medium–high small vessel disease burden (L-SVD and H-SVD, respectively).

		L-SVD group	H-SVD Group

		*N* (%)	*N* (%)
# Patients		31 (100)	29 (100)
Central oculomotor features^a^	Spontaneous/evoked nystagmus of a central type	5 (16.1)	7 (24.1)
	Broken smooth pursuit	11 (35.5)	13 (44.8)
	Hypometric saccades	4 (12.9)	8 (27.6)
	Hypermetric saccades (overshooting)	1 (3.2)	2 (6.9)
	Square waves jerks	2 (6.4)	4 (13.8)
	Head shaking nystagmus with central features	2 (6.4)	4 (13.8)

*^a^More findings than cases are listed due to patients with multiple central oculomotor features*.

**Table 3:** Patients with central oculomotor abnormalities among different degrees of Fazekas’ scale.

	Fazekas 1	Fazekas 2	Fazekas 3

	*N*/tot (%)	*N*/tot (%)	*N*/tot (%)
# Patients	31/60 (51.6)	22/60 (36.6)	7/60 (11.6)
Patients with central oculomotor abnormalities	20/31 (64.5)	17/22 (77.3)	6/7 (85.7)

Dizziness Handicap Inventory scores varied greatly and on average were slightly higher in the H-SVD group (14.9 and 33.4 in L-SVD and H-SVD, respectively); however, this did not quite reach statistical significance (*p* = 0.054).

## Discussion

White matter hyperintensities on brain MRI are common according to a normal aging, and they can affect diverse CNS regions (11, 12). The white matter lesions burden positively correlates with age, with a higher prevalence of severe SVD in patients older than 76 years (8). A significant involvement of the brain by SVD is linked with the development of geriatric syndromes (1, 2) and can truly represent a clinical entity able to cause dizziness and falls in the elderly (6, 9). Eye movement control pathways for saccades and pursuit involve ascending and descending fibers between frontal and parietal cortex, basal ganglia, brainstem, and cerebellum (13).

Changes in vestibular function are also present with increasing age—including degeneration of peripheral vestibular structures (14), neuronal loss in the vestibular nuclei and their cortical projections (15), and a higher prevalence of BPPV (16). A cross-sectional study in a population of elderly patients demonstrated a 9% prevalence of unrecognized BPPV; the multivariate analysis showed also that patients with unrecognized BPPV were more likely to have reduced daily living activities and depression (17). The primary aim of this study was to survey, with neurotological investigations, possible causes of dizziness in a group of SVD patients referred to a neurovascular clinic. Interestingly, despite the presence of SVD, we found a high incidence of pathological peripheral vestibular findings (35.0% of the total), particularly in patients with low-grade SVD; on the contrary, despite SVD being positively correlated with age, a high SVD burden was not related to a higher incidence of vestibular abnormalities. Furthermore, the lack of clear difference in DHI scores between L-SVD and H-SVD groups confirms that, in case of high burden, SVD can cause unexplained dizziness *per se*: the majority of patients with severe SVD have unexplained dizziness, in turn severe SVD can affect quality of life similar to a peripheral vestibular disorder. All these aspects agree with our previous study, in which the dizziness was explainable mostly in patients with lower SVD burden; on the contrary, SVD-related dizziness featured mostly in patients with higher SVD burden (9).

Our second research question was to determine whether increasing SVD could produce oculomotor abnormalities of diagnostic value, presumably by involvement of these pathways. Our study, however, did not show a significantly different prevalence of central oculomotor features between the L-SVD and H-SVD groups. Furthermore, no consistent pattern of abnormal oculomotricity was detectable. In agreement with Pinkhardt and colleagues, it may be possible that the deficits in oculomotor and cognitive functioning probably depend more on which fibers are hit by SVD rather than the amount of fibers affected (18).

This study has one major limitation, given by the lack of correlation of our results with the topography of brain SVD lesions, more than with the total SVD burden. However, it shows a different perspective for clinicians who routinely see elderly patients with dizziness or unsteadiness with white matter lesions on MRI: these kinds of symptoms, in case of mild SVD, are often associated with pathological vestibular findings. This is of relevance both to vascular neurologists and to gerontologists, because a considerable percentage of dizzy patients with mild SVD are more likely to suffer from an undiagnosed peripheral vestibular disorder. These kinds of pathologies are usually treatable either by therapeutic maneuvers (BPPV) (19) or by vestibular rehabilitation programs (uncompensated vestibular loss) (20); this would help also in reducing the risk of falls and depression.

In summary, in this study, we report that high load of SVD on imaging, absence of clinical/laboratory evidence of vestibular disease and, as reported in our previous paper, subtle gait and postural abnormalities (9), seems a way to support a diagnosis of SVD-related dizziness in elderly patients. However, in terms of diagnostic value, central oculomotor alterations do not seem to delineate any helpful consistent pattern. On the contrary, a complete neurotological evaluation seems to be crucial: the high prevalence of peripheral vestibular findings highlights the importance of recognizing any underlying, potentially treatable, cause of dizziness, especially those represented by peripheral vestibular disorders.

## Ethics Statement

This study was carried out in accordance with the hospital protocols. All subjects gave written informed consent in accordance with the Declaration of Helsinki. The protocol was approved by the Pisa University Ethic Committee.

## Author Contributions

NC: conception and design of the study, evaluation of patients, statistical analysis, drafting the article, critical revision, and final approval of the manuscript. MM: conception and design of the study, evaluation of patients, critical revision, and final approval of the manuscript. EN: contribution to the study design, evaluation of patients, critical revision, and final approval of the manuscript. NG: contribution to the study design, evaluation MRI scans, critical revision, and final approval of the manuscript. AC: contribution to the study design, review of clinical and laboratory findings, critical revision, and final approval of the manuscript.

## Conflict of Interest Statement

No potential conflicts of interest were disclosed. Our institution did not receive any payment or services from a third party for any aspect of the submitted work. All authors do not declare any financial relationships with entities that could be perceived to influence, or that give the appearance of potentially influencing. All authors declare no patents or copyrights, whether pending, issued, licensed, and/or receiving royalties relevant to the work. All authors declare no relationships or activities that readers could perceive to have influenced, or that give the appearance of potentially influencing, what you wrote in the submitted work.
